# Switching nanoscale temperature fields with high-order plasmonic modes in transition metal nanorods†

**DOI:** 10.1039/d3ra06649e

**Published:** 2023-11-24

**Authors:** Kenji Setoura, Mamoru Tamura, Tomoya Oshikiri, Takuya Iida

**Affiliations:** a Department of Mechanical Engineering, Kobe City College of Technology Kobe Hyogo 651-2194 Japan setoura@kobe-kosen.ac.jp; b Division of Materials Physics, Graduate School of Engineering Science, Osaka University Toyonaka Osaka 560-8531 Japan; c Research Institute for Light-induced Acceleration System (RILACS), Osaka Metropolitan University Sakai Osaka 599-8570 Japan; d Research Institute of Multidisciplinary Research for Advanced Materials, Tohoku University Sendai Miyagi 980-8577 Japan; e Research Institute for Electronic Science, Hokkaido University Sapporo Hokkaido 001-0021 Japan; f Department of Physics, Osaka Metropolitan University Sakai Osaka 599-8531 Japan

## Abstract

Depending on the photoirradiation conditions, metal nanostructures exhibit various plasmonic modes, including dipolar, quadrupolar, and hexapolar modes. This work demonstrates numerically that these high-order plasmonic modes can be used to switch nanoscale temperature distributions during the plasmonic heating of a manganese (Mn) nanorod. The key feature of Mn is its low thermal conductivity. Generally, when noble metal nanostructures are used for plasmonic heating, the nanostructure surface will be almost isothermal regardless of the order of the excited plasmonic modes because of the high thermal conductivity of noble metals, *e.g.*, the thermal conductivity of gold is 314 W m^−1^ K^−1^. However, unlike noble metals, Mn has a significantly lower thermal conductivity of 7.8 W m^−1^ K^−1^. Due to this lower thermal conductivity, the distinct spatial characteristics of the high-order plasmonic modes can be transcribed clearly into nanoscale temperature fields, which are achieved by generating polarization currents by high-order plasmons within the nanorod. These findings strongly suggest that high-order plasmonic modes hold significant potential for the advanced and precise manipulation of heat generation at the nanometer scale in thermoplasmonics.

## Abstract 2

Nanoscale temperature distributions can be dramatically switched by high-order plasmonic modes in transition metal nanorods.

## Graphical abstract

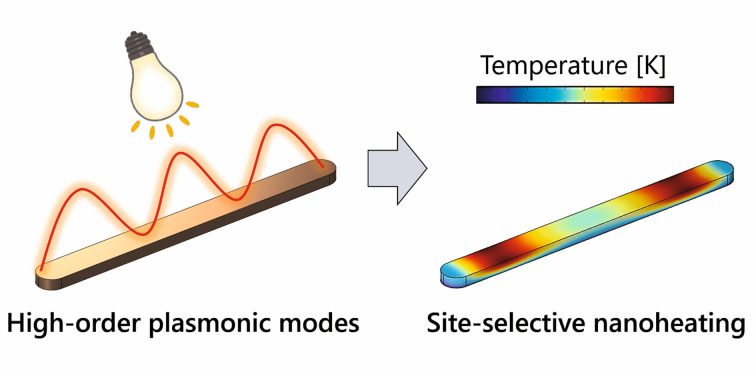

## Introduction

Plasmonic nanoparticles have two primary functions: electric field enhancement and photothermal conversion.^1,2^ Although the optical excitation of the lowest mode, known as the dipolar mode or the 1st mode, forms the foundation, high-order plasmonic modes, such as the quadrupolar mode (the 2nd mode) and the hexapolar mode (the 3rd mode), have received considerable attention for their capability to enhance and modulate interactions between light and matter or both.^3–7^ In photothermal conversion, the utilization of high-order plasmonic modes is still in the developmental stage. One of the primary challenges in employing these modes in thermoplasmonics is the diffusive nature of the heat transfer. Typically, the photothermal conversion of plasmonic nanoparticles involves three main steps:^8^ (i) optical excitation of localized surface plasmons (LSP), (ii) heat generation by the Joule heating effect resulting from polarization currents, and (iii) a localized temperature increase through heat diffusion or conduction. During step (ii), the nanoparticle's polarization currents and heat power density exhibit intrinsic nanoscale spatial distributions corresponding to the specific excited plasmonic modes.^9^ However, in most cases of plasmonic heating employing noble metal nanoparticles, these distinct spatial distributions tend to diminish owing to heat diffusion.^10^ As a result, the nanoparticle surface becomes nearly isothermal, regardless of the order of the excited plasmonic modes.^11^

To overcome these limitations and effectively utilize high-order modes in thermoplasmonics, a straightforward solution involves employing plasmonic materials with low thermal conductivity (*k*) to minimize heat dissipation. While the widely recognized plasmonic material, gold (Au), possesses an extremely high thermal conductivity of *k*_Au_ = 314 W m^−1^ K^−1^, certain transition metals and their nitrides exhibit plasmonic optical properties^12^ and low thermal conductivities. For example, manganese (Mn) possesses a low thermal conductivity of *k*_Mn_ = 7.8 W m^−1^ K^−1^,^13^ and titanium nitride (TiN) exhibits a thermal conductivity of *k*_TiN_ = 29 W m^−1^ K^−1^.^14^ These materials present promising alternatives for achieving efficient thermoplasmonic processes owing to their combined plasmonic characteristics and reduced heat dissipation.^15,16^ In our previous study, we conducted numerical simulations to demonstrate that the dipolar plasmonic mode can be transcribed into the temperature field through polarization currents during the plasmonic heating of a TiN nanoring with a diameter of 200 nm.^17^ Notably, we observed temperature differences (Δ*T*) exceeding 100 K on the surface of the nanoring, which were dependent on the specific irradiation conditions.

**Scheme 1:**
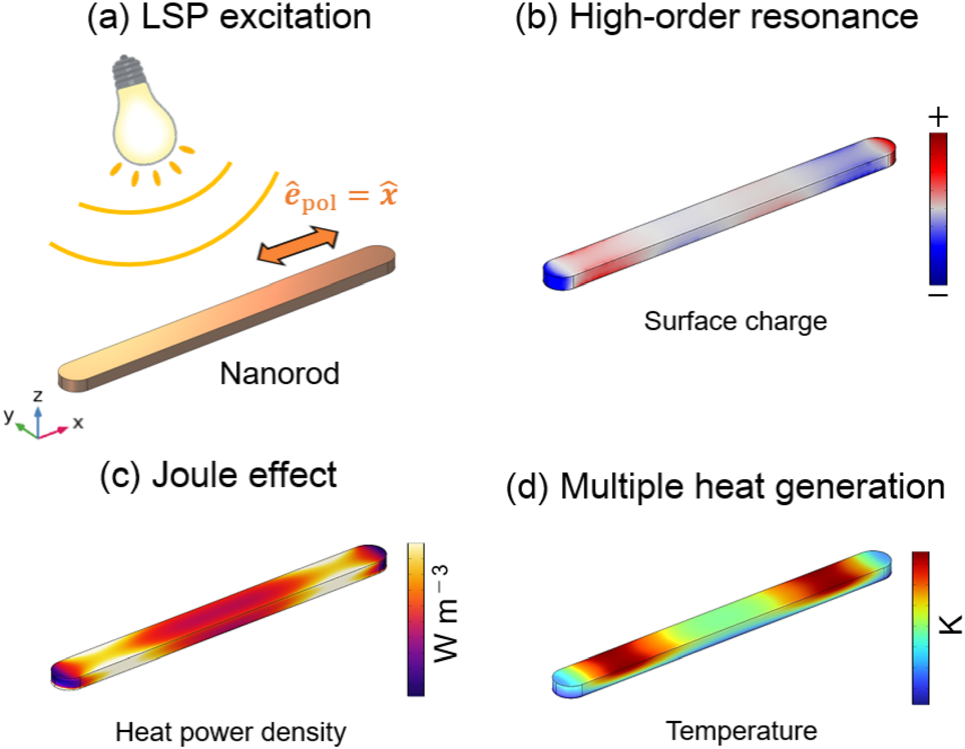
(a) LSP excitation of the Mn nanorod. (b) Polarization formation by the high-order plasmonic mode. (c) Photothermal conversion *via* the Joule heating effect. (d) Local temperature increase at multiple positions.

Along this line, it would be possible to transcribe high-order plasmonic modes into nanoscale temperature fields with much higher spatial resolutions using Mn instead of TiN, owing to its low thermal conductivity. The concept of this study is illustrated in Scheme 1. The process is as follows. (i) In Scheme 1a, a Mn nanorod with a high aspect ratio is illuminated with a linearly polarized plane wave, effectively exciting localized surface plasmons (LSPs). (ii) Scheme 1b shows that high-order plasmonic modes are excited, manifesting distinctive spatial characteristics at a specific wavelength. (iii) The Joule heating effect generates the heat power density within the nanorod, as depicted in Scheme 1c. Notably, the excited plasmonic modes directly influenced the spatial distribution of the heat power density.^9^ (iv) Finally, in Scheme 1d, selected regions along the nanorod experience localized heating contingent upon the specific excited plasmonic mode. By following this sequence, this study aims to elucidate the correlation between the plasmonic modes, heat generation, and the resulting spatial temperature distribution in Mn nanorods. Although Mn can be oxidized easily under atmospheric conditions, there are few reports on the fabrication of plasmonic Mn nanoparticles.^18^ Consequently, it is possible to fabricate Mn nanostructures using electron beam lithography techniques.

In this study, we numerically calculated the electric field, surface charge density, heat power density, and temperature field of Mn nanorods using the finite element method (FEM). As a result, it was revealed that the nanoscale temperature fields could be switched dramatically using the 5th and 7th plasmonic modes along the longer axis of the nanorod, and the hot spots on the nanorods were split into two or three depending on the plasmonic modes. This intriguing phenomenon holds tremendous potential for advanced heat generation control in thermoplasmonics. These findings contribute to advancing and refining techniques for precisely manipulating and harnessing thermal energy at the nanoscale.

## Results and discussion

### Numerical modelling and characterization of LSPR

**Fig. 1:**
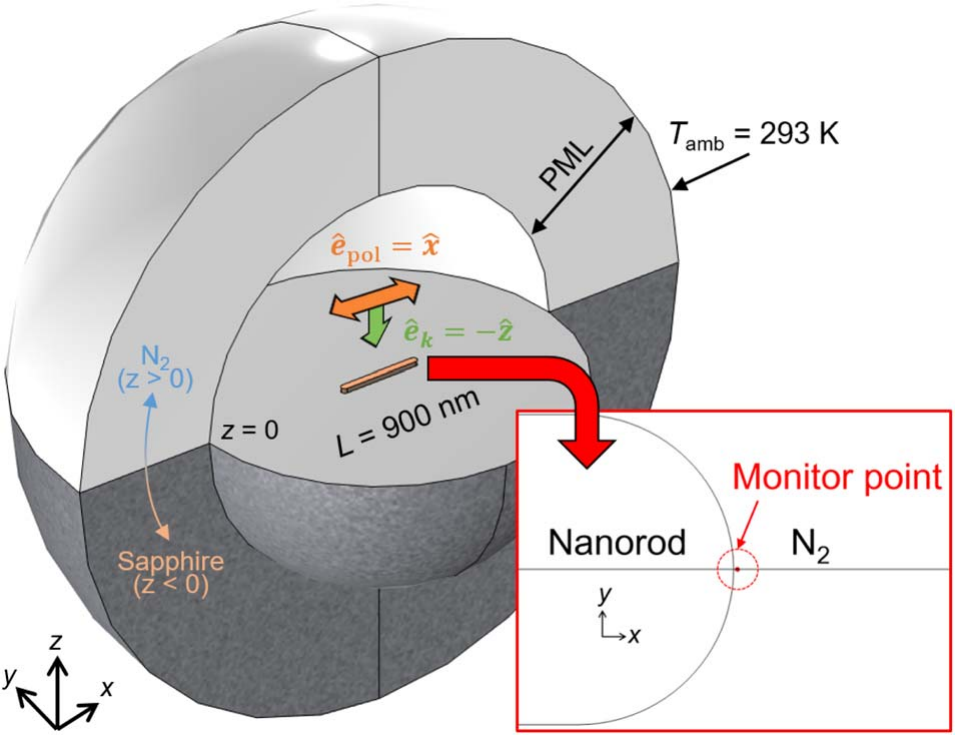
Geometry for the numerical simulations. A Mn nanorod with a length of 900 nm is placed on a sapphire substrate and exposed to a nitrogen environment. The Mn nanorod is illuminated with a linearly polarized plane wave from above. Inset: The electric field amplitude (|***E***|) was monitored at the red dot location to evaluate near-field spectra.

While dynamic temperature changes resulting from the pulsed optical heating of metal nanostructures are intriguing,^19^ we focused on studying the steady-state temperature fields under continuous illumination in this work, primarily for the sake of simplicity. Fig. 1 shows the geometry of the numerical simulations. A Mn nanorod with a length of 900 nm, a width of 80 nm, and a thickness of 30 nm was placed on a sapphire substrate and exposed to a nitrogen environment. The sapphire was used to enhance the temperature gradients [K m^−1^] around the nanorod owing to its high thermal conductivity (*k*_sapphire_ = 42 W m^−1^ K^−1^), and the Mn nanorod is free from oxidation in the nitrogen environment. We assumed the injection of an *x*-polarized plane wave in the *z*-direction to excite the nanorod's plasmonic modes along the longitudinal axis (L-mode). To evaluate the near-field spectra, we monitored the electric field amplitude (|***E***|) normalized by the incident amplitude (|***E***_0_|) at the location indicated in the inset of Fig. 1. This specific point was positioned 1 nm from the surface of the nanorods at *z* = 15 nm. We solved Maxwell's equations in the frequency domain and the steady-state heat conduction equations for this system using the FEM software COMSOL Multiphysics (version 6.1). Although the detailed numerical methods can be found in our previous paper,^17^ we briefly introduce them here. These equations are expressed as follows:1
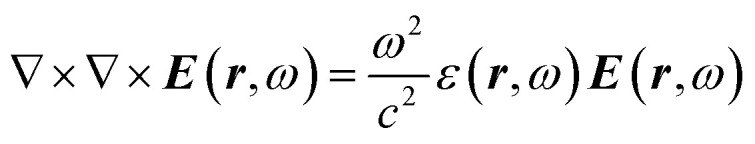
2
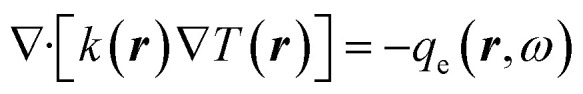
Here eqn (1) is the wave equation derived from Maxwell's equations for nonmagnetic materials, ***E*** is the electric field, *ω* is the angular frequency of light, *c* is the speed of light, *ε* is the relative dielectric function depending on the observation position ***r*** (*ε* is approximately vacuum permittivity (*ε*_0_) outside the nanorod and substrate. The value of *ε* inside the nanorod is given with the dielectric functions of Mn), eqn (2) is the heat conduction equation, *k* is the thermal conductivity, *T* is the temperature, and *q*_e_ is the volumetric heat power density arising from the Joule heating effect. The Joule-heating effect driven by an oscillating electromagnetic field at an arbitrary frequency is expressed as follows:^19,20^3
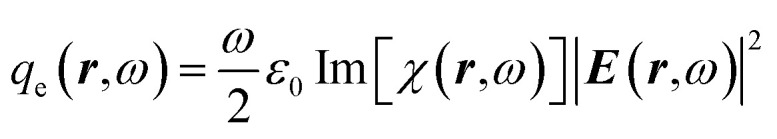
where *ε*_0_ is vacuum permittivity. As boundary conditions, the simulation system was surrounded by perfectly matched layers (PMLs) for eqn (1), and a Dirichlet boundary condition with *T*(***r*** = 3.5 μm) = *T*_amb_ = 293 K was imposed for eqn (2). The solution of eqn (1)–(3) for the system depicted in Fig. 1 provides comprehensive numerical results encompassing the plasmonic extinction spectra and spatial distributions of electric fields, heat power density generated by the Joule effect, and temperature fields. These results consider the interactions among the nanorod, the nitrogen environment, and the sapphire substrate. As clearly shown in the above equations, the intrinsic spatial characteristics of the plasmonic modes (***E***(***r***, *ω*)) can be transcribed into temperature fields (*T*(***r***)) using the heat power density (*q*_e_(***r***, *ω*)). The measured values of the relative dielectric function of Mn were taken from the literature;^21^ for comparison, the relative dielectric functions of Mn, TiN,^22,23^ and Au^24^ are summarized in the ESI Fig. S1.† Other physical constants, including the refractive indices and thermal conductivities, are summarized in Table 1.

**Table 1:** Materials properties

	Nitrogen	Sapphire	Mn
Relative dielectric function *ε*	1.0003^2^	1.77^2^	*ε*_Mn_ (*ω*)
Thermal conductivity *k* [W m^−1^ K^−1^]	0.023	42	7.8

**Fig. 2:**
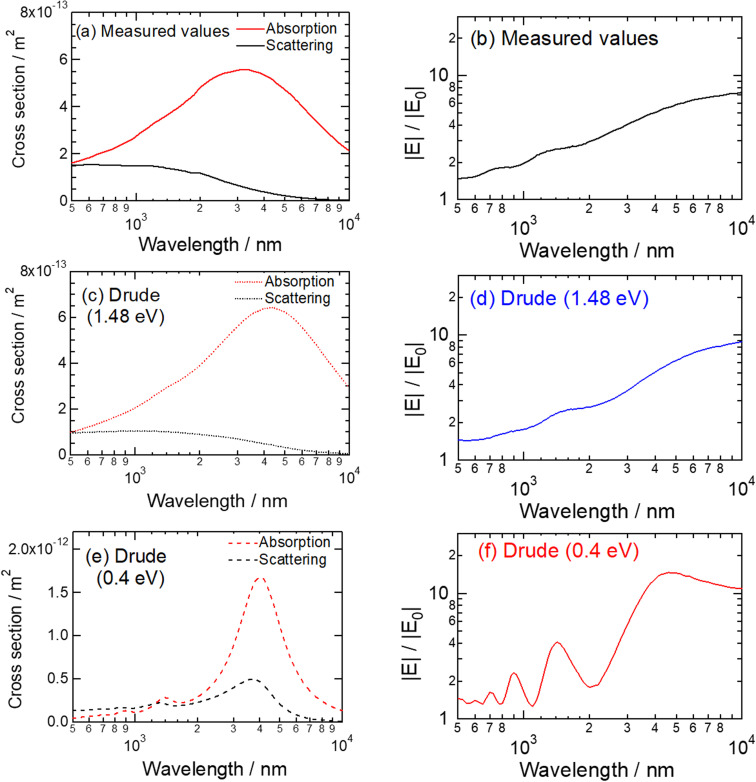
Absorption, scattering, and near-field spectra (|***E***|/|***E***_0_|) of the Mn nanorod. The measured values of the dielectric functions were used for (a) and (b). The dielectric functions calculated by the Drude model were used for (c)–(f). For (c) and (d), the damping constant of 1.48 eV was used. For (e) and (f), the damping constant of 0.4 eV was used.

First, we calculated the localized surface plasmon resonance (LSPR) spectra and the near-field spectrum of the Mn nanorod with the above numerical model, as shown in Fig. 2a and b: the evaluation methods of the LSPR spectra can be found in the literature.^17^ In Fig. 2a, while the Mn nanorod exhibited weak scattering signals across a wide spectral range, a clear LSPR peak was observed in the absorption spectrum within the wavelength region of 3 to 4 μm. While we identified the LSPR peak in Fig. 2a, no discernible peak was observed in the near-field spectrum presented in Fig. 2b, even within the wavelength range of 3 to 4 μm. Consequently, we encountered difficulties attributing specific plasmonic modes solely to these spectra. We speculate that these faint and broadened plasmonic characteristics of the Mn nanostructure may stem from the pronounced damping of Mn compared to noble metals, as exemplified by the dielectric functions shown in Fig. S1.†

To provide a quantitative characterization of the plasmonic modes within this long nanorod, we opted to utilize the dielectric functions of Mn, which were computed numerically using the Drude model rather than relying on the measured values. The following equation defines the Drude model:^4,18^4
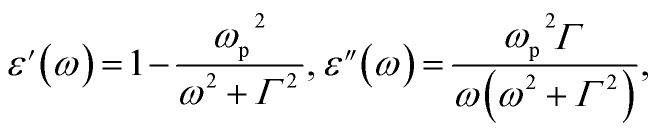
where *ω*_p_ is the plasma frequency in a vacuum, and *Γ* is the damping constant. Per the literature,^18^ plasmonic spectra from synthesized Mn nanoparticles closely matched the numerical results obtained *via* Mie theory using dielectric functions calculated by the Drude model. In that paper, the authors identified that the values *ħω*_p_ = 7.6 eV and *ħ**Γ* = 1.48 eV were suitable for Mn. We calculated the LSPR and near-field spectra of the nanorods by employing these two parameters with the Drude model, as shown in Fig. 2c and d. Remarkably, the LSPR spectra in Fig. 2c closely resemble those in Fig. 2a. Furthermore, the near-field spectra in Fig. 2b and d exhibit nearly identical behaviors. Consequently, we can confidently confirm that the Drude model provides a suitable representation of the dielectric functions of Mn for numerical simulations of plasmonic nanostructures.

Now that we have established the validity of the Drude model, we can explore the plasmonic modes within the Mn nanorod by systematically decreasing *ħ**Γ* while keeping *ħω*_p_ constant at 7.6 eV. It is worth noting that reducing *ħ**Γ* is a well-established and rigorous method for characterizing plasmonic modes, particularly in cases where plasmonic spectra are complex and broad, as reported in the literature.^4^ We systematically reduced *ħ**Γ* in steps of 0.1 eV, starting from 1.4 eV, and subsequently calculated the dielectric functions, LSPR, and near-field spectra. The dielectric functions calculated using the Drude model for each damping constant are summarized in Fig. S2.†

Finally, we identified distinct spectral peaks corresponding to high-order plasmonic modes at *ħ**Γ* = 0.4 eV, as shown in Fig. 2e and f. In Fig. 2e, the scattering and absorption spectra displayed pronounced peaks around 4 μm, which we attribute to the dipolar mode, as explained later in this section. Several peaks emerged in the scattering and absorption spectra in wavelength regions of 1.2 to 1.4 μm and 800 to 900 nm. These multiple peaks appear to be characteristic features of high-order plasmonic modes observed in relatively longer nanorods.^25^ In the near-field spectrum, as shown in Fig. 2f, we distinctly observed four peaks at 4600, 1425, 925, and 700 nm wavelengths, aligning with the LSPR peaks evident in the far-field spectra.

We could straightforwardly assign the plasmonic modes associated with each peak wavelength from these near-field spectra by considering the photon energy ratio. The peak wavelengths at 4600, 1425, 925, and 700 nm correspond to photon energies of 0.27, 0.87, 1.34, and 1.77 eV, respectively. Given that the strongest peak (0.27 eV) can be attributed unequivocally to the dipolar mode (the 1st mode), we can infer the order of the high-order plasmonic modes by examining the photon energy ratios relative to the 1st mode. These ratios are listed in Table 2.

**Table 2:** The high-order plasmonic modes

	Wavelength	Photon energy	Ratios
1st mode	4600 nm	0.27 eV	1.0
3rd mode	1425 nm	0.87 eV	3.2
5th mode	925 nm	1.34 eV	5.0
7th mode	700 nm	1.77 eV	6.6

The ratios presented in Table 2 allow us to assign the spectral peaks observed in Fig. 2f to specific plasmonic modes. Accordingly, the peaks at 1425, 925, and 700 nm can be attributed to the 3rd (hexapolar), 5th (decapolar), and 7th (14-polar) modes, respectively. It is worth noting that the 2nd (quadrupolar) and 4th (octapolar) modes are forbidden because of the symmetry between the incident light and plasmons.

**Fig. 3:**
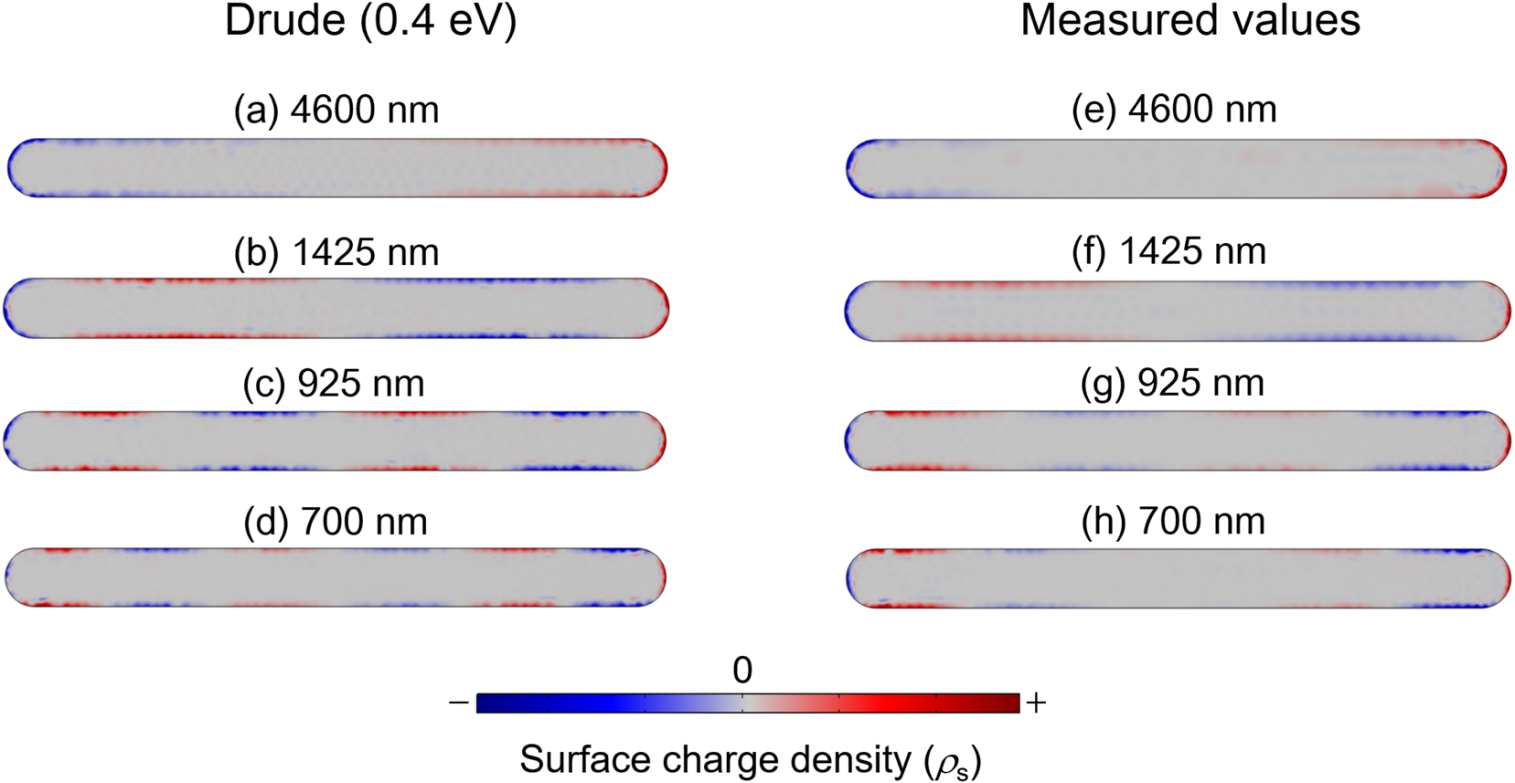
Surface charge density of the Mn nanorod. The dielectric functions calculated by the Drude model were used for the left panel. The measured values of the dielectric functions were used for the right panel.

We calculated the surface charge density (*ρ*_s_ [C m^−2^]) on the Mn nanorod to further support our mode assignments. Gauss's law should be applied in the following form to compute the *ρ*_s_ on the boundary between the plasmonic nanostructure and the surrounding environment:

5 *ρ*_s_ = ***n***·(***D***_2_ − ***D***_1_)

where ***n*** is the outward normal vector, ***D***_1_ and ***D***_2_ are the electric field displacement inside and outside the Mn nanorods, respectively. These calculations were performed for the four wavelengths summarized in Table 2, and the dielectric functions used were those obtained from the Drude model with *ħω*_p_ = 7.6 eV with the *ħ**Γ* of 0.4 eV. The results are shown in Fig. 3a–d, illustrating the charge density patterns corresponding to the dipolar (a), hexapolar (b), decapolar (c), and 14-polar (d) modes at each wavelength.^3^ Based on these patterns, we can confidently conclude that the high-order plasmonic modes in the Mn nanorod have been characterized successfully using both the near-field spectra and the charge density patterns obtained with the dielectric functions calculated by the Drude model.

However, for more realistic simulations of plasmonic heating in the Mn nanorod, it is advisable to use the measured values of the dielectric functions of Mn, as reported in the literature.^21^ Even though there were no clear peaks in the near-field spectrum presented in Fig. 2b, we calculated the charge density patterns at specific wavelengths (4600, 1425, 925, and 700 nm) using the measured values of the dielectric functions of Mn. The results are shown in Fig. 3e–h. The patterns observed are dipolar (e), hexapolar (f), decapolar (g), and 14-polar (h). By comparing Fig. 3a–d with Fig. 3e–h, we observe that the polarization patterns are similar at each wavelength. Therefore, it is reasonable to assume that high-order plasmonic modes can be excited using the measured values of the dielectric functions of Mn obtained from the literature. However, the spectral peaks may not be distinguishable in Fig. 2a and b.

Before delving into the calculations of plasmonic heating of Mn nanorod, it's essential to address the significant difference in refractive indices between the nitrogen environment (*n*_nitrogen_ = 1.0003) and the sapphire substrate (*n*_sapphire_ = 1.77). When this difference is exceptionally large, plasmonic modes can split between the top and bottom of the nanostructure. This phenomenon has been extensively studied, especially for silver nanostructures on TiO_2_ substrates (*n*_TiO_2__ = 2.5) exposed to air (*n*_air_ = 1.0003).^26^ In our prior discussions, we didn't consider plasmonic mode splitting caused by the refractive index disparity between the nitrogen environment and the sapphire substrate. To address this, we conducted additional calculations for the plasmonic far- and near-field spectra of the Mn nanorod placed on borosilicate glass (*n*_glass_ = 1.52) and TiO_2_ substrates (*n*_TiO_2__ = 2.5) in the presence of a nitrogen environment. We also calculated the plasmonic spectra of the Mn nanorod floating in a homogeneous nitrogen environment for comparison. Please note that the measured values of the dielectric functions of Mn were used. The results are summarized in ESI Fig. S3–S5.† In summary, we observed no plasmonic mode splitting between the top and bottom of the Mn nanorod, even though the electric field amplitude was slightly higher at the bottom due to the high refractive index of the sapphire substrate. These findings lead to a safe conclusion: the anisotropy of plasmonic modes along the *z*-direction is negligible. This is primarily because the Mn nanorod's thickness (*t* = 30 nm) is relatively thin. According to existing literature, plasmonic mode splitting is more frequently observed in structures with heights of at least 100 nm on TiO_2_ substrates.^26^

### 2D mapping of |***E***|/|***E***_0_|, heat power density, and temperature

In this section, we will calculate the electric field, the heat power density (*q*_e_), and the temperature field around the Mn nanorod for the 1st to the 7th plasmonic modes at a constant excitation power density of 2 MW cm^−2^. Notably, the measured values of the dielectric functions of Mn were used. Consequently, these high-order plasmonic modes can be experimentally excited upon the successful fabrication of Mn nanorods.

**Fig. 4:**
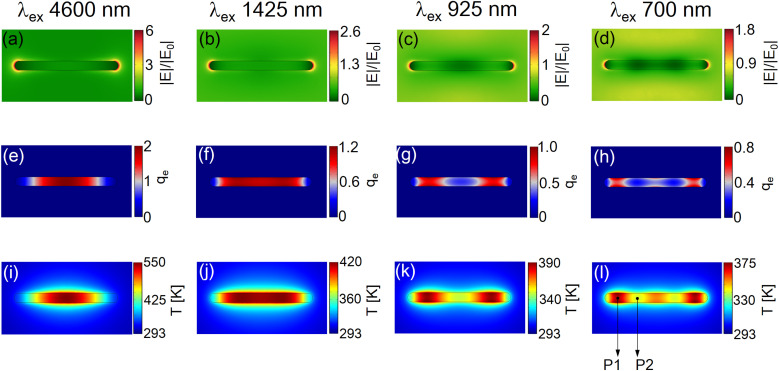
2D mapping of the electric field (a–d), the heat power density (e–h), and the temperature field (i–l) around the Mn nanorod at the excitation wavelengths of 4600, 1425, 925, and 700 nm under the constant excitation power density of 2 MW cm^−2^: these mappings were plotted on the *x*–*y* plane at *z* = 15 nm. Note that the unit for the *q*_e_ in (e–h) is (×10^18^ W m^−3^).

Fig. 4a–d show the electric field amplitude normalized by the incident amplitude on the *x*–*y* plane at *z* = 15 nm. This *z* value is half the thickness of the Mn nanorods. Despite the substantial difference in the refractive index between the nitrogen environment and the sapphire substrate, our analysis revealed negligible anisotropy in the electric field and heat power density along the *z*-direction inside and on the nanorod surface, as demonstrated in ESI Fig. S6.† Consequently, the selected *z* value was found to be reasonable. As shown in Fig. 4a, the 1st plasmonic mode (4600 nm) exhibits the highest electric field enhancement, reaching a value of approximately 6, particularly at the edges of the nanorod. For the 3rd (1425 nm), 5th (925 nm), and 7th (700 nm) modes, plasmonic hotspots were also observed at the same locations, albeit with lower degrees of enhancement ranging from 2.6 to 1.8, as shown in Fig. 4b–d. Remarkably, in Fig. 4c and d, distinct vibrational structures can be observed within the nanorod. These structures manifested as periodic alternations between bright and dark regions along the longitudinal axis of the nanorods. These periodic structures directly reflect the vibrational modes associated with high-order plasmonic modes along the long axes of the nanorods.^25^

Based on these electric fields, we calculated the heat power density (*q*_e_) resulting from polarization currents *via* the Joule heating effect. The heat power density distributions are shown in Fig. 4e–h; the unit used in these figures is (×10^18^ W m^−3^). For example, the maximum value of the heat power density in Fig. 4e is 2 × 10^18^ W m^−3^. Upon exciting the 1st mode at 4600 nm, we observed the concentration of *q*_e_ at the center of the nanorod, as depicted in Fig. 4e. This spatial pattern is consistent with observations found in relevant literature when the 1st mode is excited.^9^ When the 3rd mode was excited at 1425 nm, the spatial distribution of the heat power density (*q*_e_) exhibited broadening along the long axis of the nanorod, as illustrated in Fig. 4f. Interestingly, we observed that *q*_e_ became localized in multiple locations within the nanorod, as illustrated in Fig. 4g and h. Specifically, *q*_e_ is split into two or three regions, depending on the order of the excited plasmonic modes. When comparing Fig. 4c (electric field) and Fig. 4g (*q*_e_), it becomes apparent that the high electric field intensity regions align with the areas where *q*_e_ is concentrated in the nanorod. Conversely, the low electric field intensity regions correspond to relatively smaller values of *q*_e_. This correlation between the internal electric field and *q*_e_ can be explained by eqn (3), which describes the Joule heating effect. Therefore, it can be concluded that the specific plasmonic mode predominantly determines the nanoscale spatial distribution of heat power density. Similarly, the spatial distribution of the *q*_e_, which is split into three regions in Fig. 4h, corresponds to the vibrational structure observed in the internal electric field within the nanorod in Fig. 4d. This correspondence further highlights the interplay between the plasmonic modes, the electric field distribution, and the resulting heat power density within the Mn nanorod.

Finally, we calculated the steady-state temperature distributions around the Mn nanorod as shown in Fig. 4i–l: note that these 2D temperature mappings were plotted on the *x*–*y* plane at *z* = 30 nm. This *z*-value corresponds to the interface between the nanorods and the nitrogen environment. Our simulations indicated markedly different surface temperatures for the nanorods owing to the significant thermal conductivity disparity between the nitrogen environment and the sapphire substrate, at *z* = 0 nm (nanorod–sapphire interface) and *z* = 30 nm (nanorod–nitrogen interface), as illustrated in ESI Fig. S6.† In practical applications, the surface temperature of nanorods at *z* = 30 nm is crucial. Therefore, we focused our analysis on the temperature distributions at this specific *z*-value, as shown in Fig. 4i–l. As clearly can be seen, the nanoscale temperature distributions (Fig. 4i–l) inherited the spatial patterns of the *q*_e_ (Fig. 4e–h). Notably, while the hotspot is singular and centered within the nanorod under the excitation of the 1st and the 3rd modes, it splits into two or three distinct hotspots depending on the excited plasmonic modes. This observation indicates that by simply changing the excitation wavelength using plasmonic nanorods of a constant size, we can effectively switch the temperature distribution at the nanometer scale among the three distinct patterns. Despite the inherent diffusive nature of heat transfer at the nanometer scale, we conclude that it is possible to shape nanoscale temperature fields using high-order plasmonic modes. This capability is facilitated by the low thermal conductivity of Mn, which allows the preservation and translation of the unique spatial characteristics of these modes into distinct temperature distributions.

To summarize, including the results presented in Fig. 4, the *q*_e_ tends to become more delocalized within the nanorod and occasionally splits into multiple spots along the longitudinal axis as the order of the plasmonic mode increases. Further investigations are required to unravel the intricate spatial distributions of *q*_e_ when high-order plasmonic modes are excited in the future.

### Power dependence upon the excitation of the 7th mode at 700 nm

We evaluated the excitation power dependence in the temperature calculations to assess the thermal conductivity contribution quantitatively. For this analysis, we specifically focused on the excitation of the 7th mode at 700 nm because *q*_e_ was observed to be highly delocalized within the nanorods, indicating a tendency towards a more uniform temperature field. We considered three different thermal conductivities for the nanorod: *k*_Mn_ = 7.8 W m^−1^ K^−1^, *k*_TiN_ = 29 W m^−1^ K^−1^, and *k*_Au_ = 314 W m^−1^ K^−1^. The relative dielectric function was fixed at Mn. Although replacing the dielectric function of Mn with TiN or Au is feasible, such substitutions would significantly alter the plasmonic mode at a specific wavelength, complicating the evaluation of excitation power dependence. Therefore, our analysis focused solely on changing the thermal conductivities while keeping the dielectric function constant to maintain consistency when evaluating the excitation power dependence. In the calculation, we evaluated the maximum temperature difference (Δ*T*) on the nanorod surface, which was determined by subtracting the highest temperature at location P1 from the lowest temperature at location P2, as depicted in Fig. 4l.

**Fig. 5:**
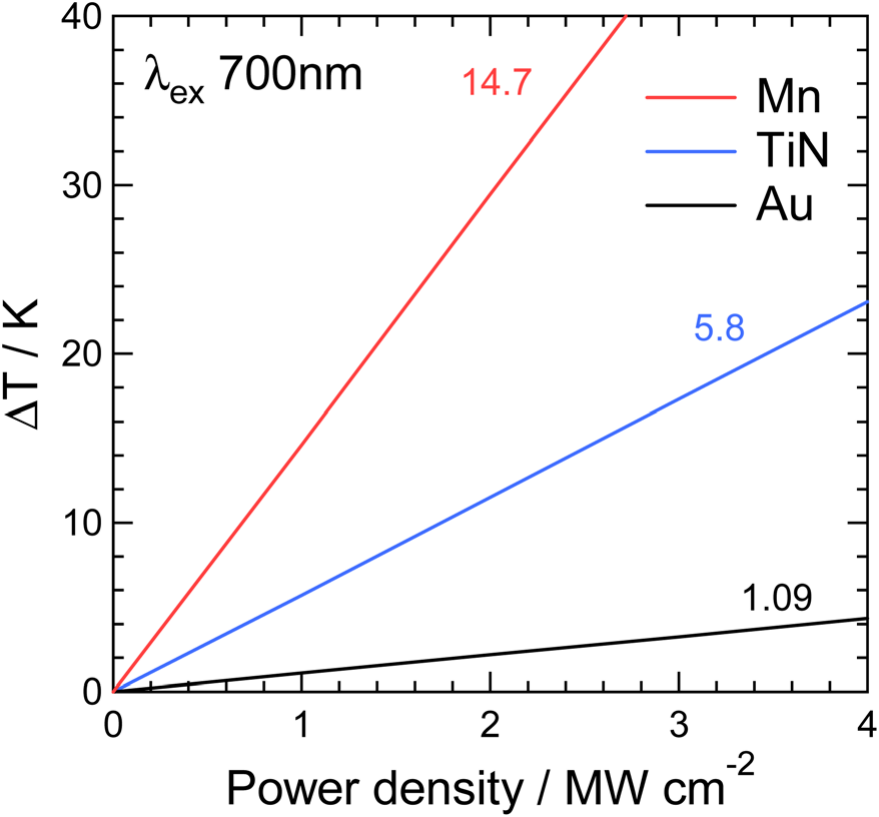
Power dependence of the maximum temperature difference (Δ*T*) upon exciting the 7th plasmonic mode at 700 nm for the three materials, Mn, TiN, and Au.

Fig. 5 shows Δ*T* as a function of the excitation power density for the three materials. For all materials, Δ*T* increased linearly with the power density. The slopes of the three straight lines are presented in Fig. 5 as 14.7, 5.8, and 1.09 for Mn, TiN, and Au, respectively. These slope ratios reflect the bulk thermal conductivities of Mn, TiN, and Au. The Δ*T* for Au is very small, indicating that nanoscale temperature shaping is almost impossible with Au nanostructures.

In contrast, the largest Δ*T* was observed for Mn, highlighting its exceptional plasmonic nanoscale temperature shaping capability. This finding underscores the superior performance of Mn in achieving precise and controlled temperature modulation at the nanoscale, although Mn is oxidized under atmospheric conditions. TiN, with an intermediate slope of 5.8, offers practical advantages for nanoscale temperature shaping. Because TiN is free from oxidation and exhibits thermal durability at high temperatures,^27^ therefore, TiN emerges as a realistic choice for precisely controlling temperature at the nanoscale. The fabrication of fine TiN nanostructures using techniques such as electron-beam lithography^28,29^ and photolithography^15^ has been reported in several studies. These findings further support the feasibility and practicality of TiN for nanoscale temperature modulation applications.

## Conclusions

We investigated the spatial shaping of a nanoscale temperature field with the high-order plasmonic modes in Mn nanorods. Using the finite-element method, we calculated the optical heating of a plasmonic Mn nanorod illuminated by a linearly polarized plane wave. Initially, we characterized the plasmonic modes across a wide spectral range, observing the 1st, 3rd, 5th, and 7th modes at excitation wavelengths of 4600, 1425, 925, and 700 nm, respectively. Subsequently, we computed the nanoscale temperature fields resulting from the heat power density generated by the Joule heating effect upon the excitation of these high-order modes. Remarkably, the hotspots within the nanorod split into two or three regions, depending on the order of the plasmonic modes. The strong correspondence between the spatial distribution of the heat power density and the internal electric field within the nanorod led us to conclude that the spatial patterns of the high-order plasmonic modes are transcribed faithfully into the nanoscale temperature fields. Finally, we elucidated the advantages of Mn compared to TiN and Au based on the excitation power dependence. Our findings highlight the significant utility of high-order plasmonic modes in thermoplasmonics, enabling the precise control of heat generation at the nanometer scale.

## Author contributions

K. S. coordinated the project. All authors performed the numerical calculations. The manuscript was written with contributions from all the authors. All authors approved the final version of the manuscript.

## Conflicts of interest

There are no conflicts to declare.

## Supplementary Material

RA-013-D3RA06649E-s001
