# Supplementary material for: Switching nanoscale temperature fields with high-order plasmonic modes in transition metal nanorods
Source: RSC Adv. 2023 Nov 24;13(49):34489–96. doi: 10.1039/d3ra06649e (PMC10667964; doi:10.1039/d3ra06649e)
Supplement: RA-013-D3RA06649E-s001 [file RA-013-D3RA06649E-s001.pdf]

Electronic Supplementary Material (ESI) for RSC Advances.
This journal is © The Royal Society of Chemistry 2023

Electronic Supplementary Information for

Switching Nanoscale Temperature Fields with High-Order Plasmonic Modes in Transition Metal Nanorods

Kenji Setoura,*[a] Mamoru Tamura, [b, c] Tomoya Oshikiri, [d, e] and Takuya Iida [c, f]

[a.] *Department of Mechanical Engineering, Kobe City College of Technology, Kobe, Hyogo 651-2194, Japan.*

[b.] *Division of Materials Physics, Graduate School of Engineering Science, Osaka University, Toyonaka, Osaka, 560-8531, Japan*

[c.] *Research Institute for Light-induced Acceleration System (RILACS), Osaka Metropolitan University, Sakai, Osaka 599-8570, Japan*

[d.] *Research Institute of Multidisciplinary Research for Advanced Materials, Tohoku University, Sendai, Miyagi 980-8577, Japan*

[e.] *Research Institute for Electronic Science, Hokkaido University, Sapporo, Hokkaido 001-0021, Japan*

[f.] *Department of Physics, Osaka Metropolitan University, Sakai, Osaka 599-8531, Japan*

E-mail: setoura@kobe-kosen.ac.jp

Contents

1. The relative dielectric functions of Mn, TiN, and Au (**Fig. S1**).
2. The relative dielectric functions of Mn calculated by the Drude model (**Fig. S2**).
3. The far- and near-field spectra of the Mn nanorod placed in various environments (**Figs. S3, S4, and S5**).
4. Calculated electric field, heat power density, and temperature distribution around the Mn nanorod at an excitation wavelength of 4600 nm on the x-z plane (**Fig. S6**).

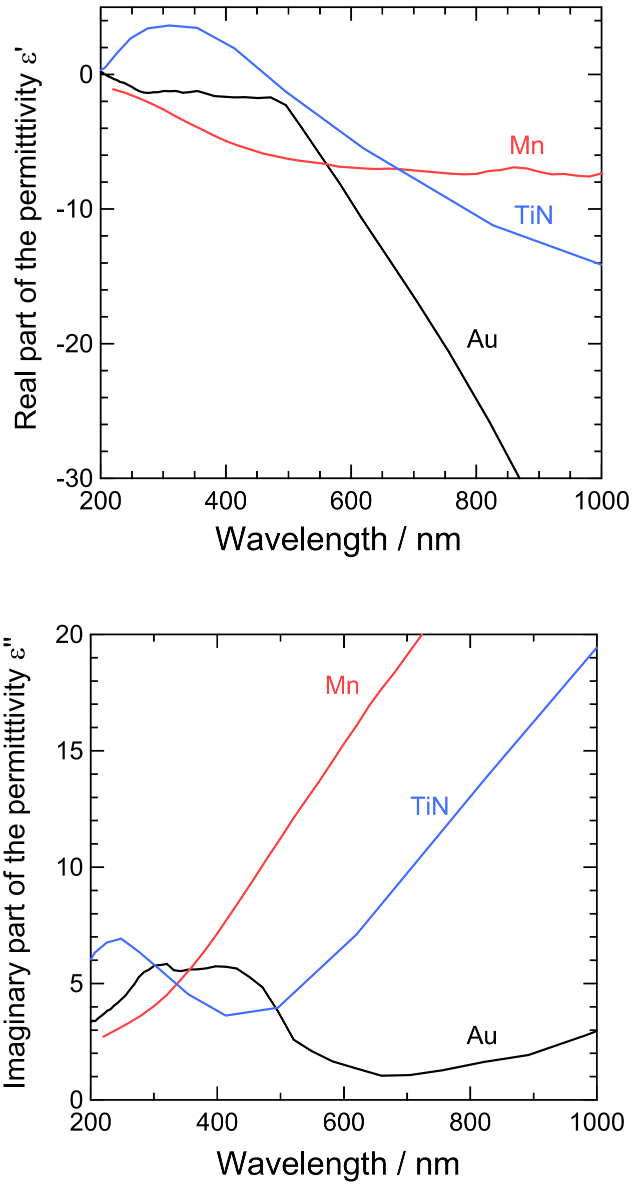

Fig. S1 The relative dielectric function of Mn [1], TiN [2, 3], and Au [4] reproduced from the literatures.

1. M. Querry, "Optical Constants of Minerals and Other Materials from the Millimeter to the Ultraviolet", Report No. ADA192210, U.S. Army Chemical Research, Development & Engineering Center, Aberdeen Proving Ground, MD **1987**.
2. J. Pflüger and J. Fink, in Handbook of optical constants of solids II, Academic Press, pp. 293–310, **1991**.
3. J. Pflüger, J. Fink, W. Weber, K. P. Bohnen, G. Crecelius, "Dielectric properties of TiCx, TiNx, VCx, and VNx from 1.5 to 40 eV determined by electron-energy-loss spectroscopy", *Phys. Rev. B*, 30, 1155-1163, **1984**.
4. P. B. Johnson and R. W. Christy. "Optical constants of the noble metals", *Phys. Rev. B*, 6, 4370-4379, **1972**.

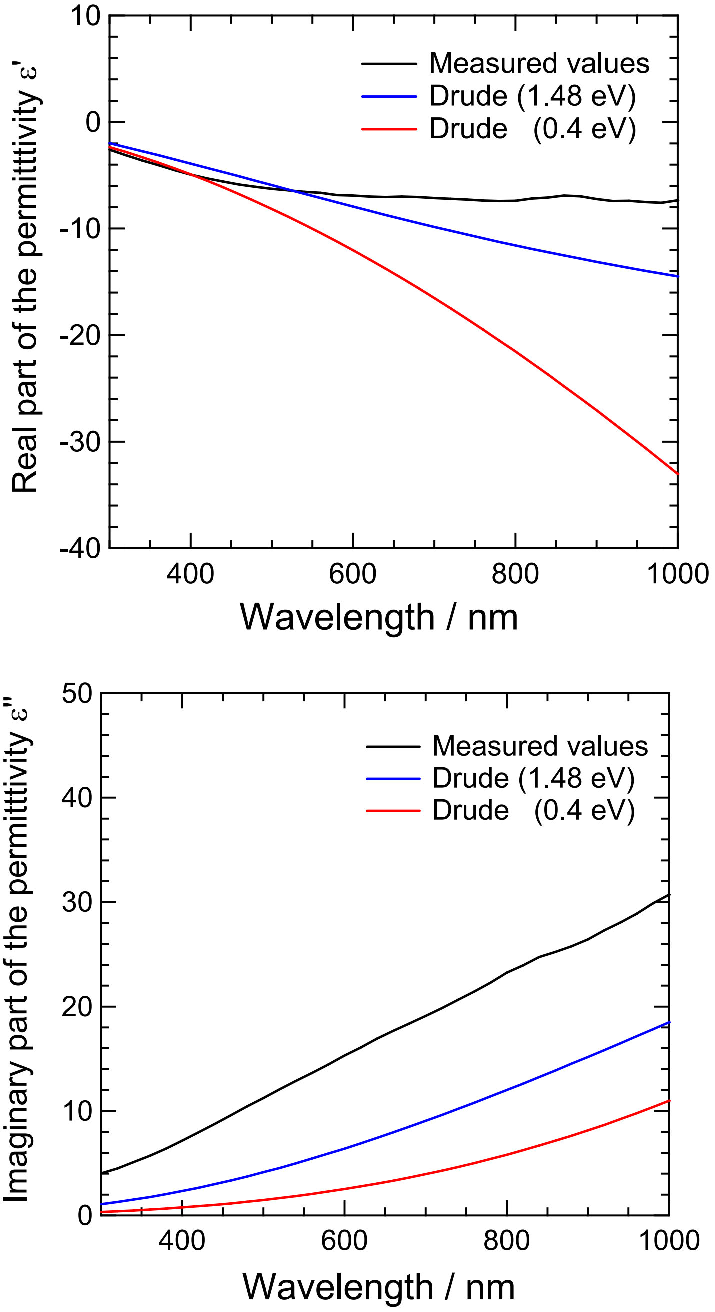

Fig. S2 The relative dielectric function of Mn reproduced from the literature [1] and calculated by the Drude model [2].

1. M. Querry, "Optical Constants of Minerals and Other Materials from the Millimeter to the Ultraviolet", Report No. ADA192210, U.S. Army Chemical Research, Development & Engineering Center, Aberdeen Proving Ground, MD **1987**.
2. Oleg A. Yeshchenko et al., "Optical properties of sol-gel fabricated Mn/SiO_2 nanocomposites: Observation of surface plasmon resonance in Mn nanoparticles", *Applied Surface Science*, 254 (**2008**) 2736–2742.

To investigate whether plasmonic mode splitting occurs at the top and bottom of the nanorod, we calculated the plasmonic far- and near-field spectra of the Mn nanorod in a homogeneous nitrogen environment, as well as when placed on borosilicate glass, sapphire, and TiO_2 substrates and exposed to the nitrogen environment. The refractive indices of the employed materials are as follows: n_{nitrogen} = 1.0003, n_{glass} = 1.52, n_{sapphire} = 1.77, and n_{TiO_2} = 2.5. The numerical procedures are detailed in the main article. Please note that the measured values of the dielectric functions of Mn were used. First, we focused on the far-field spectra. In Fig. S3a and S3b, we present the scattering and absorption spectra of the Mn nanorod in various environments. Fig. S3a shows a decrease in scattering intensity with an increasing refractive index of the substrate, although no distinct spectral peak is observed. Conversely, localized surface plasmon resonance (LSPR) peaks are evident in the absorption spectra, as displayed in Fig. S3b. The LSRP peak demonstrates red-shifts with an increasing refractive index of the substrate. This refractive index dependency on the surrounding environment is a typical behavior observed in the optical properties of plasmonic nanostructures, as reported in numerous studies [e.g. M. A. Garcia, *J. Phys. D: Appl. Phys.* 45, (**2012**), 389501]. These findings clearly indicate that the refractive index of the substrate influences the plasmonic mode in the Mn nanorod. However, for a more detailed analysis of plasmonic mode splitting, a thorough examination of the near-field spectra is necessary.

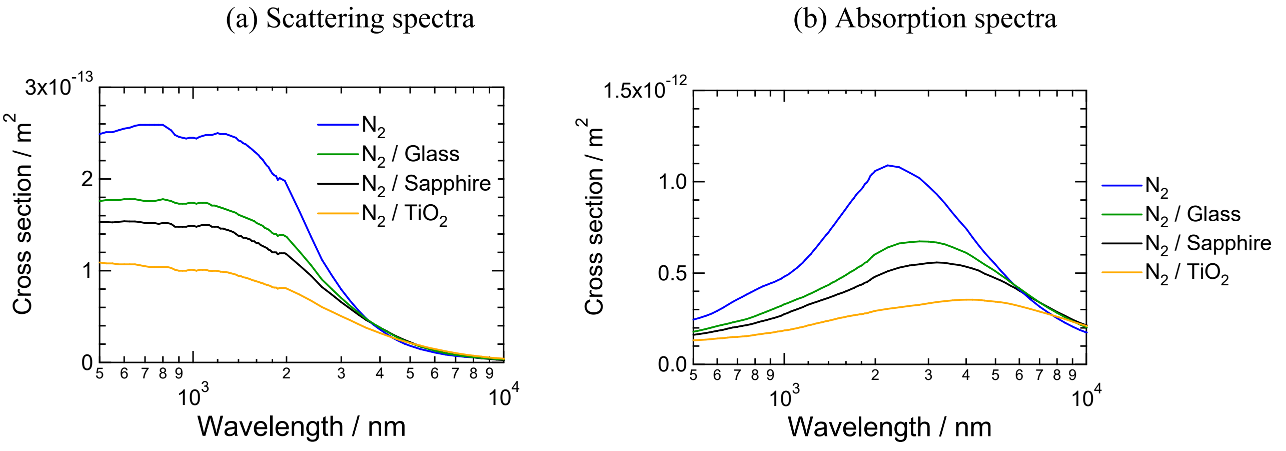

Fig. S3 Scattering (a) and absorption spectra (b) of the Mn nanorod floating in the homogeneous nitrogen environment, the Mn nanorod placed on various substrates and exposed to the nitrogen environment.

To assess plasmonic mode splitting, we monitored the electric field amplitude (|***E***|) normalized by the incident amplitude (|$\boldsymbol{E}_0$|) at three specified locations as indicated in the inset of Fig. S4. The top is at (x, y, z) = (451 nm, 0 nm, 29 nm), the middle is (451 nm, 0 nm, 15 nm), and the bottom is (451 nm, 0 nm, 1 nm), respectively.

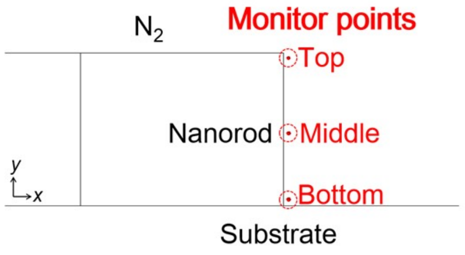

Fig. S4 Monitor points for evaluation of the near field spectra at the top, middle, and bottom of the Mn nanorod.

Fig. 5a to d display the near-field spectra of the Mn nanorod in various environments. In Fig. S5a, the field enhancement values were almost the same at the top (red open triangles) and the bottom (blue open squares) because the Mn nanorod was placed in a homogeneous environment. However, the enhancement values at the middle were slightly lower than those at the top and the bottom. This occurred because the electric field can be enhanced at the edges of nanostructures.

Next, let's focus on the near-field spectra of the Mn nanorod placed on the substrates shown in Fig. S5b to d to discuss the anisotropy of the plasmonic modes at the top and the bottom. In all the spectra, the enhancement values at the bottom were greater than those at the top. This disparity arose because the refractive indices of the substrates are much higher than that of the nitrogen environment. However, across all the spectra in Fig. S5b to d, the enhancement curves at every location (top, middle, and bottom) exhibited almost identical behavior. This clearly indicates that the anisotropies of the plasmonic modes at the top and the bottom of the Mn nanorod are negligible. According to the literature [I. Tanabe et al., *Nano Lett.*, **2012**, 12, 5418.], plasmonic mode splitting can be observed for silver nanostructures with typical heights of about 100 nm placed on TiO_2 substrates and exposed to air. In this article, we used a much thinner Mn nanorod with a thickness of 30 nm; therefore, the plasmonic mode splitting at the top and the bottom was practically unobservable. Additionally, the strong damping of Mn may have played a role in suppressing the plasmonic mode splitting.

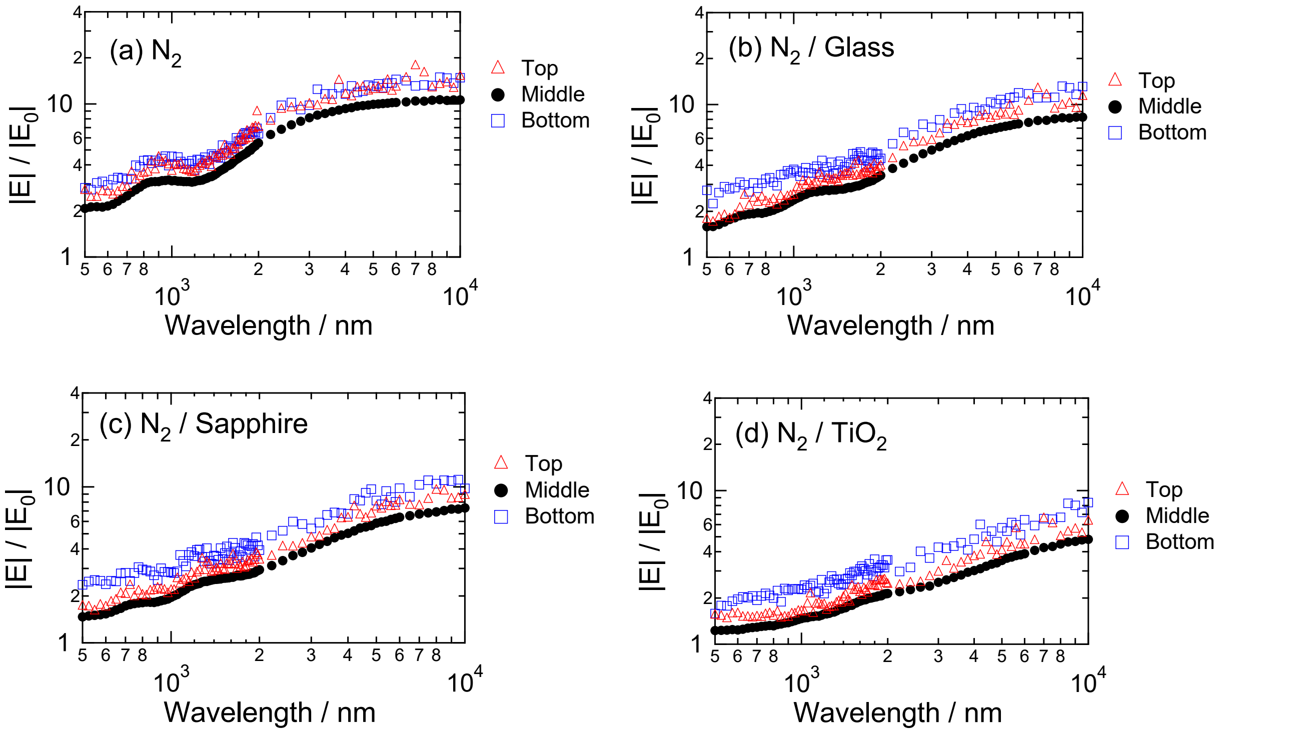

Fig. S5 Near-field spectra of the Mn nanorod in various environments: (a) floating in the homogeneous nitrogen environment. (b)-(c) placed on various substrates and exposed to the nitrogen environments.

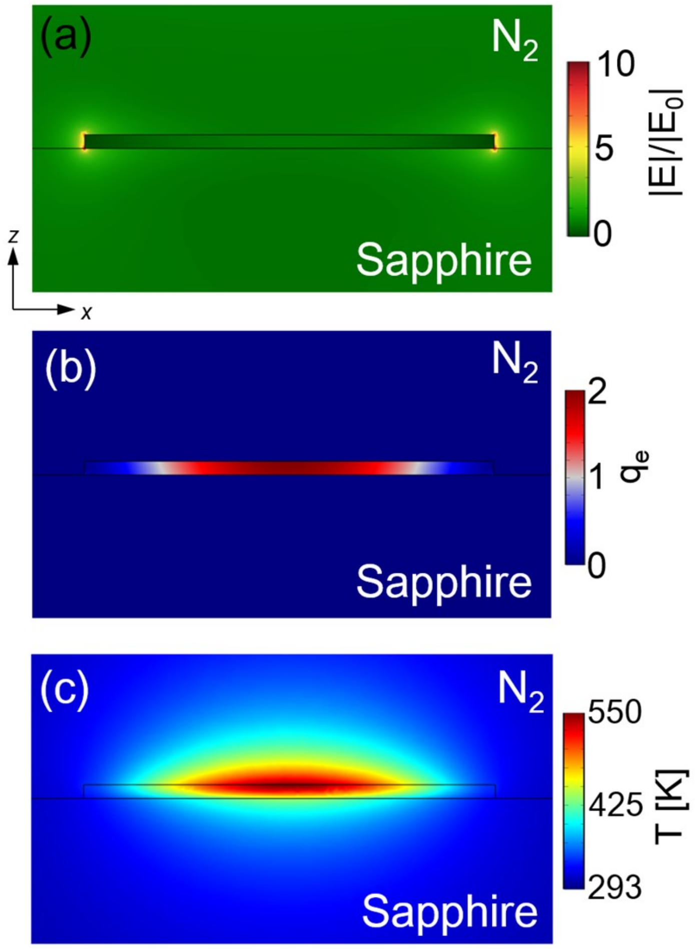

Fig. S6 2D mapping of the electric field (a), the heat power density (b), and the temperature field (c) around the Mn nanorod at the excitation wavelength of 4600nm under an excitation power density of 2 [MW cm^{-2}]: these mappings were plotted on the x-z plane at y = 0 nm. Note that the unit for the q_e in (b) is ($\times 10^{18}$ [W m^{-3}]).
